# Stereotactic MRI-guided Adaptive Radiation Therapy (SMART) for Locally Advanced Pancreatic Cancer: A Promising Approach

**DOI:** 10.7759/cureus.2324

**Published:** 2018-03-14

**Authors:** Elaine Luterstein, Minsong Cao, James Lamb, Ann C Raldow, Daniel A Low, Michael L. Steinberg, Percy Lee

**Affiliations:** 1 Department of Radiation Oncology, University of California, Los Angeles

**Keywords:** locally advanced pancreatic cancer, sbrt, mri-guided adaptive radiotherapy, smart

## Abstract

Locally advanced pancreatic cancer (LAPC) is characterized by poor prognosis and low response durability with standard-of-care chemotherapy or chemoradiotherapy treatment. Stereotactic body radiation therapy (SBRT), which has a shorter treatment course than conventionally fractionated radiotherapy and allows for better integration with systemic therapy, may confer a survival benefit but is limited by gastrointestinal toxicity. Stereotactic MRI-guided adaptive radiation therapy (SMART) has recently gained attention for its potential to increase treatment precision and thus minimize this toxicity through continuous real-time soft-tissue imaging during radiotherapy. The case presented here illustrates the promising outcome of a 69-year-old male patient with LAPC treated with SMART with daily adaptive planning and respiratory-gated technique.

## Introduction

Locally advanced pancreatic cancer (LAPC) has traditionally been treated with first-line single-agent chemotherapy or conventionally fractionated chemoradiotherapy over a period of five to six weeks, with characteristically poor prognosis due to unresectability of a disease in which long-term survival is achieved almost exclusively through curative resection. While newer therapeutic agents such as nab-paclitaxel and FOLFIRINOX have improved the efficacy of these standard-of-care treatments [1], survival outcomes remain poor, and studies are ongoing to evaluate the benefit of various radiotherapy treatment modalities in combination with emergent systemic therapies.

The LAP07 trial determined that conventionally fractionated chemoradiotherapy is able to achieve survival outcomes similar to those of chemotherapy alone but introduces additional treatment toxicities [2]. The predominant benefit of chemoradiotherapy was improved local control, which suggested a sustained role for radiotherapy through an intensified radiotherapy regime that would increase dose and reduce toxicity.

Stereotactic body radiation therapy (SBRT) is an innovative treatment modality notable for its shorter treatment course (one to two weeks), which is beneficial for systemic therapy integration and allows for minimal interruption of chemotherapy. A review of locally advanced pancreatic cancer patients in the National Cancer Database juxtaposed SBRT with conventionally fractionated radiotherapy (CFRT) and found that SBRT was a viable radiotherapy modality that conferred a significant overall survival (OS) benefit (13.9 months vs 11.6 months for CFRT) [3]. A related study, in which patients without metastases after two cycles of gemcitabine were treated with SBRT, demonstrated a median OS of 20 months and a local control rate of 85% at median follow-up time (21 months) [4]. The study targeted patients most likely to benefit from local therapy and addressed previous trials that found chemoradiation to have no apparent survival advantage, with initial chemotherapy response used to exclude patients with rapid metastatic development after induction therapy.

SBRT, however, presents several challenges, whereby toxicity remains an issue at higher doses and treatment efficacy is compromised by lower doses. A phase II multi-institutional trial sought to characterize the gastrointestinal toxicity of SBRT, delivered in five fractions to patients without metastases after up to three cycles of gemcitabine, and reported acute and late grade ≥2 gastrointestinal toxicity rates of 2% and 11%, respectively, for a patient cohort with a median OS of 13.9 months and a one-year local control of 78% [5]. In a large single-institution series of LAPC and borderline resectable pancreatic cancer patients, a similar one-year local rate of 78% was reported for unresected patients, with a median OS of 15 months for LAPC patients, and the rate of grade ≥3 toxicity was 7% [6].

MRI-guided radiation therapy (MRgRT) optimizes the delivery of hypofractionated radiotherapy, allowing SBRT to achieve higher doses while avoiding damage to critical structures. Endoscopic ultrasound-guided placement of fiducial markers in linac-based SBRT allows for target position verification and location tracking during treatment, but this approach fails to account for inter-fractional changes of surrounding radiosensitive tissue. MRgRT can be utilized to spare radiosensitive normal tissue adjacent to the treatment target: MRgRT counters the limitations of poor soft-tissue resolution of available cone-beam computed tomography (CT) daily imaging with superior soft-tissue visualization and addresses stochastic inter-fractional changes with on-board adaptive planning.

The following LAPC case is illustrative of the unique capabilities of stereotactic MRI-guided adaptive radiation therapy (SMART), whereby treatment was delivered with respiratory-gating and adaptive plans were implemented for each of the radiotherapy fractions.

## Case presentation

**Figure 1:**
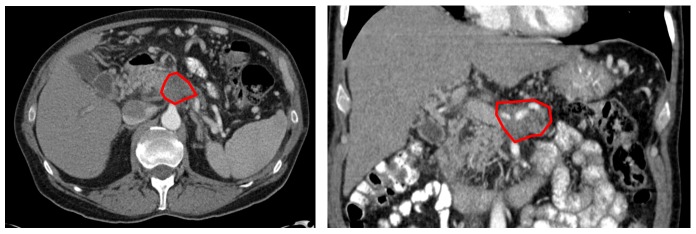
Diagnostic computed tomography with visible pancreatic body lesion.

The patient is a 69-year-old man with no family history of cancer and a prior medical history significant only for hypertension and irritable bowel syndrome. He presented with three months of abdominal pain radiating to his back and a weight loss of 12 pounds; CT imaging demonstrated a 4.7 x 3.1 cm mass within the pancreatic body, with circumferential encasement of the proximal hepatic artery, superior mesenteric artery, and left gastric artery, and occlusion of the superior mesenteric vein (Figure 1). He was diagnosed with clinical stage III (T4N1M0) locally advanced pancreatic adenocarcinoma with mucinous features on April 28, 2016. At the time of diagnosis, his CA 19-9 tumor marker level was 5645, and he was started on FOLFIRINOX systemic therapy. On August 15, 2016 the patient was seen in consultation at an outside institution, where he was offered surgical management with distal or total pancreatectomy and arterial and venous reconstruction (modified Appleby procedure), but he sought a second opinion. After eight cycles of chemotherapy, he presented for evaluation for radiotherapy on September 8, 2016. His primary pancreatic body lesion had decreased to 3.8 x 3.3 cm, his CA 19-9 had dropped to 112, and his Karnofsky performance status was 100.

SMART was recommended at a dose of 40 Gy in five fractions (alpha/beta ratio of 10 biologically effective dose [BED] = 72), and the patient received definitive SBRT to the pancreatic body over a period of 13 days (9/20/16 – 10/3/16). The gross tumor volume (GTV) was expanded by 3 mm to obtain the planning target volume (PTV), and respiratory gating was performed with a 3 mm margin with deep inspiratory breath-hold. Normal tissue constraints were defined as follows: duodenal loop and stomach V35 Gy < 0.5 cc; spinal cord and left and right kidneys maximum 12.5 Gy; skin maximum 39.5 Gy, V36.5 < 10 cc; liver ≥ 1000 cc < 15 Gy. The prescribed dose constraints were more stringent than reported objectives in similar studies and represent the ideal target and normal tissue dosimetry, while adaptive plan constraints were V35 Gy < 1 cc in the duodenum and stomach and a maximum of 20 Gy for the spinal cord.

**Figure 2:**
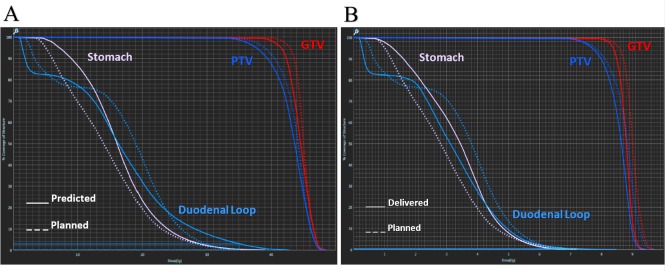
A) Dose-volume histogram comparing the treatment plan dosimetry with predicted doses based on fraction 4’s anatomy. The predicted plan significantly violates the prescribed dose constraints for the duodenal loop and stomach and inadequately covers the treatment target. B) Dose-volume histogram illustrating the adapted (delivered) treatment plan. The radiation dose to the organs at risk has been greatly reduced, while PTV coverage has been increased. (GTV - Gross tumor volume; PTV - Planning target volume)

Prior to each treatment, the initial planned treatment dose was predicted onto the patient’s current anatomy to ascertain whether the treatment plan was optimal for that day’s specific anatomy (Figure 2, Table 1). The planned doses prioritized normal tissue over PTV coverage, with adaptive planning deemed necessary if the predicted target coverage fell below that of the plan. Adaptive planning constraints were not as conservative as the prescribed objectives, and the delivered doses were well within the delineated adaptive constraints.

**Table 1:** Comparison of the planned, predicted, and delivered (adaptive) doses for fraction 4 in relation to the prescribed dose constraints. (Doses reported per fraction.) (PTV - Planning target volume; OAR - Organ at risk)

Structure	Prescribed	Planned	Predicted	Delivered
PTV: Pancreas	≥ 95% at 8 Gy	91.99	87.54	89.62
OAR: Duodenal Loop	≤ .5 cc at 7 Gy	0.28	5.96	0.96
OAR: Stomach	≤ .5 cc at 7 Gy	0.55	1.13	0.13
OAR: Spinal Cord	Max 2.5 Gy	2.37	2.11	2.52
OAR: Right Kidney	Max 2.5 Gy	2.34	2.11	1.88
OAR: Left Kidney	Max 2.5 Gy	2.71	2.31	2.52
OAR: Liver	≥ 1000 cc < 3 Gy	2265.55	2202.82	2240.30
OAR: Skin	≤ 10 cc at 7.3 Gy	0.00	0.00	0.00
OAR: Skin	Max 7.9 Gy	7.30	7.30	7.30

**Figure 3:**
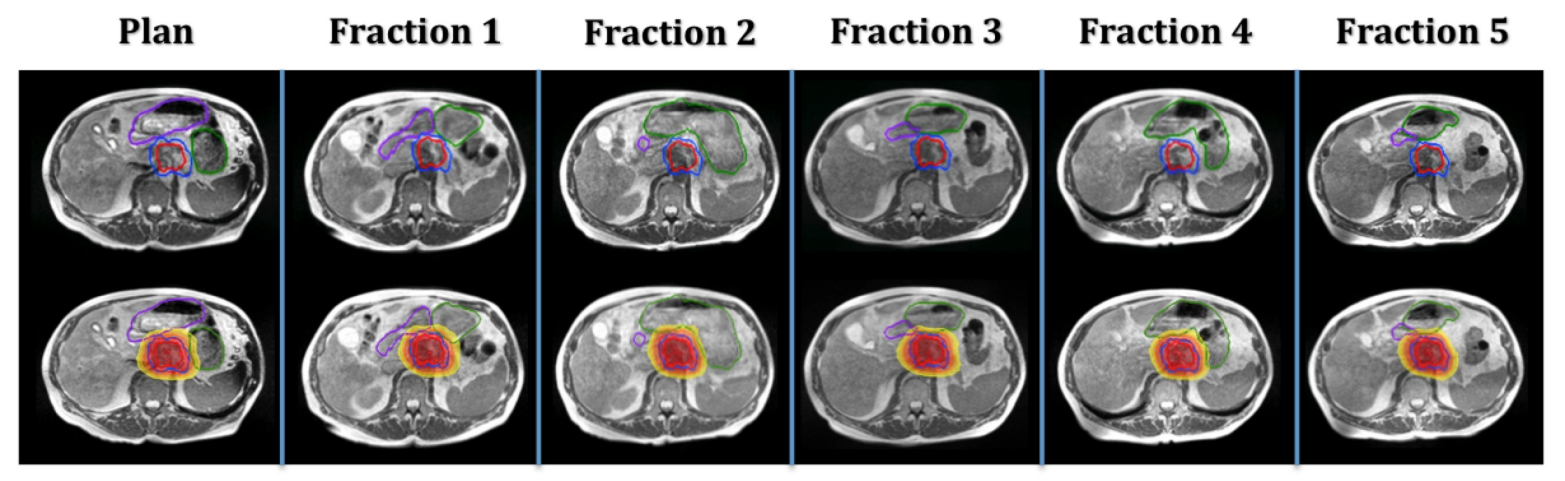
Daily positional changes and deformations of the duodenum (purple contour) and stomach (green contour).

Adaptive planning was deemed necessary for each of the five fractions due to clinically significant changes in the anatomical relationships between the tumor and normal tissue (Figure 3), which resulted in suboptimal treatment target coverage and excessive radiation of adjacent normal tissue of the stomach and duodenum.

The patient experienced bilateral shoulder discomfort with the length of treatment during the first fraction but did not suffer any adverse events during or following radiation. At the completion of treatment, his CA 19-9 level had further diminished to 55. His systemic therapy (FOLFIRINOX) was resumed after radiotherapy to achieve a total of 12 cycles, concluding on December 27, 2016. Although there is a trend towards improved survival in resected LAPC patients [6], and resection remains the only curative intent option, the patient was not interested in surgical management.

The patient continues to undergo follow-up scans every three months and continues to exhibit sustained stability of the radiated pancreatic lesion. Follow-up CT scans on June 27, 2017 demonstrated lung nodules that were suspicious for metastasis but were found to have no avidity on positron emission tomography (PET). PET/CT on November 29, 2017 revealed mildly avid, slowly progressing lung nodules, and the patient opted for surveillance. The patient remains asymptomatic and was most recently evaluated at his 14-month follow-up.

## Discussion

The value of MRgRT is demonstrated by the reported patient, who received a BED of 72 delivered through SMART with no significant toxicities and minimal treatment discomfort with the length of treatment. With continued local control at 16 months post radiotherapy and 21 months since diagnosis, the patient is an excellent example of the benefit conferred by on-board imaging and adaptive planning. The patient’s response to treatment points to a favorable outcome in the context of LAPC treatment and emphasizes the value of MRgRT in abdominal soft-tissue radiation.

The total treatment length was clinically feasible and well-tolerated by the patient, with the minimal shoulder discomfort during the first fraction in large part attributable to difficulty with inspiratory breath-hold, which diminished with each subsequent fraction. The adaptive planning process, which includes MRI imaging, contouring, re-optimization, and plan evaluation and quality assurance, took between 15 and 20 minutes, while the treatment delivery time ranged from 21 to 35 minutes, depending on the patient’s breath hold duty cycle. Contouring was performed by the physician the day of treatment, and the contouring inconsistencies between fractions (seen in Figure 3) highlight a limitation of adaptive planning due to time constraints. However, while there are discrepancies in contouring between fractions, the stomach and duodenum had identical dose constraints, and gastrointestinal tract protection was the main goal. This did not significantly affect dosimetry decisions regarding the necessity of plan adaptation, and the dosimetry constraints were based on absolute volumes rather than percentages, which additionally minimized the effects of the contouring changes. There remains a trade-off between contouring precision and administering treatment on a clinically deliverable timeline, but development of MR-Linac and auto-segmentation of normal structures are expected to reduce adaptive planning time and treatment length as the process undergoes further optimization.

The success of MRgRT adaptive planning in sparing normal tissue, demonstrated here, may have important implications in treatment dose escalations, which have been shown to improve survival outcomes. A multi-institutional study presented at the American Society for Radiation Oncology (ASTRO) in 2017 determined that patients treated with BED > 70 achieved a higher rate of overall survival than patients with a BED < 70, with 66% of the former cohort treated with adaptive portions that allowed for the safe administration of the higher doses [7]. MRgRT allows for delivery of higher BEDs without impacting surrounding tissue, and a prospective, multi‐institutional phase II study of SMART for LAPC is already planned to validate successful early retrospective results of safe and effective dose escalation with MRgRT in pancreatic cancer treatment.

## Conclusions

MRgRT stands at the forefront of efforts to deliver higher doses of radiation more accurately through the use of soft-tissue imaging before and during treatment. Radiation of LAPC is hindered by severe accompanying gastrointestinal toxicity, and SMART may play an important role in achieving local control with minimal toxicity. Currently, there are plans to escalate dose further to 50 Gy in five fractions in a prospective multi-institutional study, and trials are seeking to validate that dose escalation and more robust planning will further aid the efficacy of local therapies as systemic therapies continue to improve in combating metastatic spread. Further investigation is necessary, but early results such as this case have demonstrated the potential of SMART in advancing treatment precision and safety.
